# Arsenicum-album

**Published:** 1895-09

**Authors:** 


### HOMŒOPATHIC PHYSICIAN,

A MONTHLY JOURNAL OF

HOMŒOPATHIC MATERIA MEDICA AND CLINICAL MEDICINE.

"If our school ever gives up the strict inductive method of Hahnemann, we are lost, and deserve only to be mentioned as a caricature in the history of medicine."—CONSTANTINE HERING.

Vol. XV. SEPTEMBER, 1895. No. 9.

### EDITORIAL.

ARSENICUM-ALBUM.—The last editorial was mainly devoted to the restlessness of Arsenicum. It may be called a dissertation upon restlessness. Now attention is called to the aversion to being left alone, which is one of the indications for Arsenic. Arsenicum has fear of being left alone, and fear of death. Fear of being left alone is an indication for Apis and Phosphorus. Under Arsenicum the patient fears to be left alone lest he may do himself bodily harm. A number of other remedies have fear of being left alone. A list of these may be found in Lee's *Repertory*, chapter I, page 39. Argentum-nitricum has fear of being alone.

Quite a number of remedies have desire to be alone. These remedies are given in Dr. Lee's *Repertory* at page 22 under the rubric " Aversion to Company." There is another rubric given there also, " Desire for Company." It is needless to give these remedies here, as every subscriber to THE HOMŒOPATHIC PHYSICIAN has supplied himself with a copy of the three chapters of this *Repertory* and can look for the remedies himself.

Proceeding now to other symptoms, we find that the Arsenic patient is troubled with anxiety, reminding us of Aconite. He gets anxious at three o'clock in the morning and talks incessantly of the faults of others. This complaining of others is similar to Belladonna and Lachesis. He talks incessantly ; this is similar to Lachesis and Stramonium. One of the surest indications for Lachesis is this tendency of the patient to continuous talking.

The Arsenic patient is troubled with mental anguish.

The Arsenic patient gets headache from drinking cold water. He also has headache which is relieved by cold water applied to the head. This last symptom is different from the great characteristic of Arsenic, which is aggravation from cold applications and from cold in general.

Drinking cold water not only brings on headache, but it also excites severe pains in the bowels, with diarrhœa.

Drinking cold water causes vomiting in the Arsenic patient. The smallest quantity of cold water taken into the stomach is immediately ejected. Whenever we see this symptom we should immediately scan the Arsenic provings to see if we can find the other symptoms of the patient there, too.

The Aconite patient drinks water, vomits, and declares he will die. Then he drinks and vomits again. This is one of Dr. Guernsey's key-notes.

The Phosphorus patient vomits water as soon as it gets warm in the stomach.

Kreosote, greedy drinking followed by vomiting (Dr. Hering).

Silicea, the water tastes badly and causes vomiting.

Capsicum, drinking water causes shuddering chills, thin stools, and tenesmus.

Capsicum, as soon as he drinks water he must go to stool, but passes only a little mucus.

Argentum-nitricum, water seems to run right through him.

Arsenicum, Argentum-nitricum, Cina, Croton-tiglium, and Podophyllum all have diarrhœa after drinking water.

By looking in Bell's book on Diarrhœa you will find several other remedies that have diarrhœa after drinking.

Arsenic, while drinking water, the patient bites the tumbler.

The Arsenic patient suffering from headache desires to lie with the head high. This condition, lying with the head high, is a key-note of Arsenic. The editor, in the course of his own experience, has picked up one or two notes concerning this symptom of lying with the head high. They are as follows :

Arsenicum, aggravation from lying with the head low. There are several other remedies that have this same symptom. They are, Antimon-tart., Argentum, Capsicum, China, Colch., Hepar, Lachesis, Nitrum, Nux-vomica, Puls., Spig. See THE HOMŒOPATHIC PHYSICIAN, Vol. III, page 26 (January, 1883).

Calcarea-carbonica, amelioration from lying with the head low.

Gelsemium, amelioration of dull, heavy headache by lying with the head high.

Nux-vomica, amelioration from lying with the head high.

Much more might be added upon this one indication, but that is not the purpose of the editorial. The intention is to give the notes of Dr. Lippe, with such additions as seem to be particularly important.

For full notes and differences, the reader is once more earnestly advised to read each remedy for himself, select out all the drugs that have desire to lie with the head high, and write them down, copying the exact words of the symptom as it appears in the proving.

Arsenicum has periodical headaches. This is similar to Saccharum-officinalis and Silicea. Headache once a week, Saccharum-officinalis, Silicea, and Sulphur. Sick headache every week, Sulphur. Headache every two weeks, Sulphur. Sick headache every week, worse in forehead, with backache and bearing down, Phytolacca. Headache and all other symptoms, worse every thee weeks, Magnesia-carbonica. Headache in forehead and around eyes every six weeks, Magnesia-muriatica. Headache every seven days is Dr. Lippe's key-note for Saccharum-officinalis. Headache in forehead, temples, and about root of nose is a symptom of Arsenicum.

Tenderness of the scalp to the touch is a symptom of Arsenicum. Many other remedies have this symptom under different circumstances. Thus Kali-carbonicum has it produced by an eruption, which burns and bleeds after scratching.

Arsenic has erysipelatous burning and swelling of the head. This reminds us of Lachnantes, which has burning of scalp like fire, with thirst.

It was said that the great characteristic of Aconite is fear of death, and restlessness at night, with heat and anxiety. Arsenic has some resemblance to Aconite in this respect. It has fear of death; it has the restlessness, but of a different character. We might here repeat that the restlessness of Aconite consists in tossing and tumbling about the bed, whilst that of Arsenic drives the patient out of the bed, from one bed to another, or from one chair to another. This was plainly described in this journal last September.

Arsenic, like Aconite, has anxiety, but it occurs at three o'clock in the morning, instead of continuing all night, and it is, moreover, apt to be accompanied by fever and sick stomach.

Arsenic has inflammation of the eyelids, with burning pain, which is worse on touching the inflamed parts. The eyelids are dry, and seem as if they were scratching the eyeball. The lids burn worse when touched. Arsenic is frequently indicated in the scrofulous ophthalmia of children who have tendency to "scald head."

Arsenic has dullness of hearing, especially for the human voice. This is similar to Phosphorus, Rhus-t., Sil., and Sulph.

Rhus-tox., similarly, has hardness of hearing, especially for the human voice.

Silicea, hardness of hearing for human voice, especially during full moon.

Dr. Fellger, who was an associate of Dr. Lippe, and remarkable for his wide knowledge of medicine, once gave the editor the following comments upon the above symptom:

Arsenicum, the hearing is good for all sounds except the human voice.

Ignatia, the hearing is good only for the human voice. It is diminished for all other sounds.

Coffea, the hearing is too sensitive to music.

Phosphorus and Sepia, music pains the ear.

Graphites, the hearing is better when in a noise. It is better when riding in a carriage.

There is not space to give any more of these notes this time, and so they must be laid aside for next month.

